# Identifying research priorities for health care priority setting: a collaborative effort between managers and researchers

**DOI:** 10.1186/1472-6963-9-165

**Published:** 2009-09-15

**Authors:** Neale Smith, Craig Mitton, Stuart Peacock, Evelyn Cornelissen, Stuart MacLeod

**Affiliations:** 1 Centre for Clinical Epidemiology and Evaluation, Vancouver Coastal Health Research Institute, Vancouver, BC, Canada; 2 School of Public and Population Health, University of British Columbia, Vancouver, BC, Canada; 3 National Centre for Health Economics, Services, Policy and Ethics, British Columbia Cancer Agency, Vancouver, BC and Toronto, ON, Canada; 4 British Columbia Cancer Agency, Vancouver, BC, Canada; 5 Faculty of Health and Social Development, UBC Okanagan, Kelowna, BC, Canada; 6 Provincial Health Services Authority, Vancouver, BC, Canada

## Abstract

**Background:**

To date there has been relatively little published about how research priorities are set, and even less about methods by which decision-makers can be engaged in defining a relevant and appropriate research agenda. We report on a recent effort in British Columbia to have researchers and decision-makers jointly establish an agenda for future research into questions of resource allocation.

**Methods:**

The researchers enlisted decision-maker partners from each of British Columbia's six health authorities. Three forums were held, at which researchers and decision-makers from various levels in the health authorities considered possible research areas related to three key focus areas: (1) generation and use of decision criteria and measurement of 'benefit' against such criteria; (2) identification of so-called 'disinvestment' opportunities; and (3) evaluation of the effectiveness of priority setting procedures. Detailed notes were taken from each forum and synthesized into a set of qualitative themes.

**Results:**

Forum participants suggested that future research into healthcare priority setting would benefit from studies that were longitudinal, comparative, and/or interdisciplinary. As well, participants identified two broad theme areas in which specific research projects were deemed desirable. First, future research might usefully consider how formal priority setting and resource allocation projects are situated within a larger organizational and political context. Second, additional research efforts should be devoted to better understanding and improving the actual implementation of priority setting frameworks, particularly with respect to issues of change management and the resolution of impediments to action on recommendations for resource allocation.

**Conclusion:**

We were able to validate the importance of initial areas posed to the group and observed emergence of additional concerns and directions of critical importance to these decision-makers at this time. It is likely that the results are broadly applicable to other healthcare contexts. The implementation of this research agenda in British Columbia will depend upon the ability of the researchers and decision-makers to develop particular projects that fit within the constraints of existing funding opportunities. The process of engagement itself had benefits in terms of connecting decision-makers with their peers and sparking increased interest in the use and refinement of priority setting frameworks.

## Background

As research typically involves an investment of society's limited resources, there exists at least some obligation to ensure that research activity aligns with the interests, needs and values of the larger community. Applied health services research, in particular, focuses on practical translation and uptake of research findings [1]. The knowledge transfer and exchange literature highlights that regular interaction between researchers and decisions makers is one of the most effective ways to increase the likelihood that research knowledge will be used [2-4]. Such interaction also enables each group to better understand the contexts of the other and the constraints under which each operates. It is also critical that this interaction occurs over the entire research cycle, not just in the latter stages when some notion of 'transfer' is to take place [4,5]. Given this, it would be of great value for researchers and decision-makers to spend more time developing research directions and potential research questions collaboratively.

The field of health care priority setting and resource allocation is a good example of an applied health services research field that should produce findings aligned with the needs of the end users. However, to our knowledge there has been limited input to date, at least as reported in the literature, from decision-makers in helping to identify relevant research priorities. There are good opportunities in British Columbia to make headway in this regard. Currently there are two research groups-based at the Centre for Clinical Epidemiology and Evaluation, in Vancouver, and at the British Columbia Cancer Agency -- that have health care priority setting as their primary research interest. These two groups have collaborated with each other extensively over the last five years, have attracted provincial and national level funding, and have strong relationships with senior decision-makers in all six of the BC health authorities. These decision-makers, noting current fiscal constraints which are mirrored in many other Canadian provinces and in other countries, are keen to improve priority setting and resource allocation practices and engage in relevant research of practical importance.

Our intent in the current project was to engage decision-makers in jointly developing a set of realistic, mutually appealing research priorities. This would be accomplished through a series of interactive forums, organized around known challenges in the priority setting field. We report here on the design and implementation of these forums, and the follow up work leading to future research initiatives. Our description of the processes we undertook in BC will offer insight both into how research priorities might be developed in collaborative fashion, and into specific areas which appear today to be substantive priorities - in the short- to medium-term -- for the field of health care priority setting and resource allocation.

Lomas, Fulop, Gagnon & Allen identify two approaches to developing research priorities, the technical and the interpretive [1]. Technical approaches involve the use of existing data to drive priority choice, for instance, based on the prevalence of a disease or economic burden of illness. Interpretive approaches, by contrast, employ interactive discussion among informed stakeholders to generate priorities. This may or may not include substantial background data. Our intent in the current project was to follow the interpretive model. We were able to identify in the literature a handful of examples of joint agenda setting among health researchers and other stakeholders. In what follows, we briefly describe some of these examples, which were either provincial or national in scope.

In the US, pressure from Congress and other stakeholders in the 1990s led to efforts to make processes for setting research priorities for the National Institutes of Health more explicit, and to include a wider degree of input from the public and other stakeholders [7,8]. Rosenstock, Olenek, & Wagner describe a process used by National Institute of Occupational Safety and Health (NIOSH) to establish a national research agenda for occupational health and safety [7]. An approach derived from this model was subsequently used in the Pacific Northwest region specific to the needs of agricultural workers and employers, public health agencies, and researchers [8]. In addition, O'Fallon, Wolfle, Brown, Dearry & Olden describe sixteen "Town [Hall] Meetings" conducted over a number of years by the National Institute of Environmental Health Sciences (NIEHS) [9]. They suggest that these meetings had community impact through increased education and outreach, and generated both new research and public health policy changes.

In 1991, the UK established the National Health Service (NHS) Research & Development program with the intent of making the NHS a central agency in supporting applied health research [10,11]. Lomas et al describe efforts by the NHS Service Development and Organization (SDO) branch to consult on research priority development via what they term a 'listening model', including both an expert forum and local focus groups [1]. Noting that consumers were not directly involved in this process, other researchers subsequently followed this work with more focused consultation with service users on priorities, specifically related to midwifery and nursing research [12]. This involved a series of five focus groups in locations around England.

A more recent effort in the UK has perhaps the closest resemblance to our own work. The UK seminar series, "Managing Scarcity in the NHS: Building on Theory, Learning from Practice" (2005-2007) had a similar objective to our own--engaging government leaders, healthcare professionals, and academics to discuss resource allocation challenges (though not aiming to identify researchable projects per se). Although we are not aware of peer reviewed publications yet resulting from this series, informal communication with the organizers suggests a number of key learnings. Terminology (such as "commissioning") is understood differently by the major stakeholders and so different approaches have evolved. As well, participants noted tensions between national-level decisions and local health system management needs; efforts to strengthen local capacity were seen as desirable. Further, the systematic use of explicit priority setting frameworks has had some degree of success in central institutions, such as NICE, and so there was researcher and decision-maker interest in local adaptations of such work. Perhaps most importantly, all participants realized that cost-effectiveness analysis alone would not suffice to adequately deal with the complexities of real-world decision-making.

In Canada, major federal funding agencies have collaboratively undertaken three rounds of a stakeholder consultation exercise known as "Listening for Direction." The first was conducted in 2001 [1]. It consisted of an environmental scan, five regional workshops and a national workshop with invited participants, focused on pressing issues that decision-makers expected to encounter in the medium-term (three to five years). The process was repeated a second time in 2004 [13], and the most recent iteration, involving eight partner organizations, took place in 2007 [14].

## Methods

For this project, key contacts in each of British Columbia's six health authorities were recruited as research partners. Recruitment was done purposively; many of these individuals already had long standing interests in priority setting and resource allocation in their respective organizations. The decision-maker partners were invited to attend the forums and/or to send additional interested and appropriate colleagues. Purposeful efforts were made to engage a varied mix of people from different sectors, positions and job responsibilities (including for example program managers, finance managers, Vice Presidents and one CEO). Forums were not recorded but detailed notes were taken. At least two research team members took notes at each forum; afterward these notes were combined and any differences reconciled. Notes captured both the content of the discussion as well as contextual information which was used in subsequent analysis of the forum results. A summary of the notes, including potential research questions, was circulated to decision maker participants after each forum; this allowed them to verify that their opinions were accurately captured. The forum notes were analyzed qualitatively by the lead author, in order to group potential research questions and general comments into a set of overarching key themes; a second author provided additional analysis and review of the findings, while all authors provided comments. Subsequent to the forums, researchers contacted decision-makers directly and held one-on-one meetings to elaborate on the findings and to discuss specific areas for partnering. This served as additional participant validation of the results presented here.

### The team planning forums

Three key topics were identified and ultimately implemented as focus areas animating this series of forums:

• generation of decision criteria, deployment of criteria and measurement of 'benefit' against such criteria (September 2007)

• identification of so-called 'disinvestment' opportunities in order to release resources from a given budget to shift into higher value investment areas (January 2008)

• evaluation of the effectiveness of priority setting from the perspective of ethics and economics, noting a high degree of system complexity where each decision can have multiple effects and organizational constraints play a major role (April 2008)

These choices arose out of the researchers and decision-makers' past experiences with priority setting research in health care organizations, and our own assessment of what appeared to be gaps in the literature [15,16]. Importantly, these questions served as a starting point and initial guide to structure discussions, but it was expected that other related and/or unique issues would emerge as part of the discussion process at each forum. For instance, questions related to public engagement emerged at each of the three forums. An outside researcher with subject-area expertise was recruited to facilitate discussion of each topic.

For each forum, two presentations relevant to the day's theme were delivered: one by a researcher with interests in the given topic and the second by a decision-maker with 'on the ground' experience. Following this, there was an open, facilitated discussion based on the material presented offering decision-makers the opportunity to discuss and share their own concerns. There was a subsequent facilitated discussion explicitly aimed at eliciting ideas for potential research. Each forum was evaluated using a set of standard questions, noting whether clear goals and objectives for the day were stated and met, requesting comments on the quality of the presentations, and finally participant satisfaction with the degree of interactivity. Open-ended comments and recommendations for future forums were also solicited.

The Forums took place in Vancouver (BC's largest city and major transportation hub, which allowed for the easiest access for people coming from different sites around the province). They were held at a neutral location (i.e., not at the offices of a health authority or the researchers' institutions). No pre-reading or preparation was required. Each forum was one half day in length (4-5 hours) which allowed sufficient time to engage in the discussions but also was respectful of the many other demands on decision-makers' time.

## Results

### Features of priority setting research

Forum participants emphasized three important features of future design in priority setting research: longitudinal studies, comparative studies, and inter- or multi-disciplinary studies.

Longitudinal research would examine how priority setting processes in a health care organization develop and evolve over time. For instance, such research should consider how new approaches may be successfully implemented and maintained in the organization, as well as defining which factors facilitate or hinder this. Research could also investigate growth of organizational trust over time with respect to both leadership and joint or more collaborative, explicit decision making. It is reasonable to ask if formalized approaches to priority setting are conducive to such trust-building (I:8). [Citations here are from the research team's notes from the forums. These should be read as follows: [Forum] I: [page] 8.]

Participants welcomed opportunities for comparative research, including opportunities for quasi-experimental designs, such as studies of pre-post implementation of an explicit approach to decision-making. One participant suggested that it would be interesting to know if a requirement that resource re-allocation proposals be submitted from interdepartmental or collaborative teams would result in different results then the traditional silo approach to proposal submission (I:10). Organizations have distinct cultures--some seek to be on the 'cutting edge' in implementing new approaches to care and service delivery, while others are more content to maintain standard and accepted routines. How might these differences influence priority setting and resource allocation choices? It was noted that there have been a number of Canadian experiences with formal approaches to priority setting, such as PBMA (Program Budgeting and Marginal Analysis), and that there might be lessons drawn from a methodologically rigourous synthesis of these experiences.

Interdisciplinary research can be defined as "any study or group of studies undertaken by scholars from two or more distinct scientific disciplines. The research is based upon a conceptual model that links or integrates theoretical frameworks from those disciplines, uses study design and methodology that is not limited to any one field, and requires the use of perspectives and skills of the involved disciplines throughout multiple phases of the research process" [17]. Multidisciplinarity, by contrast, involves researchers who work on a project together, but more independently and with less crossing of disciplinary boundaries [18]. The feeling of many forum participants was that the best avenues for future research would build a knowledge base, employing concepts and drawing on literatures beyond health economics. These might include healthcare ethics, organizational psychology, and the policy sciences. Quantitative methods will have a role, but there was also a recognized place for research in the established qualitative traditions such as narrative inquiry, ethnography and discourse analysis.

### Future research theme areas

Several theme areas were also identified for future research on health care priority setting--in other words, specific ideas, topics or projects that might be pursued. These can be grouped under two broad headings, acknowledging that there will be some degree of overlap between these categories (See Table 1). First, future research might usefully consider how formal priority setting and resource allocation projects are situated within a larger organizational and political context and how resulting decisions have an impact on different parts of the continuum of care and/or different organizations entirely. Second, we might advantageously devote additional research efforts to better understanding and improving the actual implementation of priority setting frameworks.

**Table 1:** Overview of research theme areas

**Theme**	**Areas for possible investigation**
Priority setting decisions in a broader context	How can formal priority setting processes best align with and complement other decision making processes?
	
	What consequential and reactive impacts result in the implementation of priority setting approaches?
	
	Can we improve the measurement of costs and benefits to account for the full range of organizational impacts?
	
	Can we assess the relative merits of implementing formal priority setting as a small scale pilot or as an organization wide mandate?

Priority setting implementation	Report on typical criteria used in formal priority setting studies and guidance about how to draft locally relevant measures for assessing spending options
	
	Understand the different ways in which decision-makers understand and apply the concept of disinvestment
	
	Explore the rhetorical and tactical choices made in 'bundling' spending options and how these affect the results of formal priority setting
	
	Identify the personal, social and organizational dimensions of how decision-makers manage conflicting role loyalties in priority setting
	
	Improve the quality and accessibility of relevant data
	
	Identify the skills and capacities needed for effectively using formal priority setting methods, as well as the related education and training requirements
	
	Provide guidance about why, when and how to engage the public in priority setting and resource allocation decisions

#### Decisions in broader context

Forum participants noted that wherever formal priority setting processes have so far been implemented in their organizations, it has inevitably been within a larger context in which many other decision system initiatives and cycles occur simultaneously. These include strategic planning (II:3), quality improvement (I:10; II:10), capital planning (III:7), the annual budget cycle (III:3), and issuing of provincial Ministry of Health directives (II:8). There is value to decision-makers in knowing where they sit in relation to the larger network or web of decision processes. Modeling or mapping these connections might be both interesting and valuable (II:7). Case study and comparative research might consider how priority setting activities 'align' with other organizational activities.

There are also potential 'ripple' effects that might be investigated--for instance, 'how do or could priority setting exercises link to, identify, or catalyze knowledge of where larger systemic change might be needed within health authorities' (I:7)?-- how might 'local solutions ... become institutional ones' (II:6)? Also, 'a research question might be, how do executive level decisions and values translate to the everyday decisions people are making on the front lines, at the service delivery level' (II:11)?

There was some doubt among participants as to whether or not the full set of outcomes and costs for prioritization is ever captured. As one example, it was noted that while 'the literature calls regularly for reductions in acute care spending to support community based care' (III:5), we much less frequently hear about efforts to do this, and even less frequently are informed about the outcomes and whether or not any savings or improved outcomes are actually realized. For instance, 'decreasing bed use among a certain population might be seen as a success and a saving in isolation, but it may simply allow the beds to be occupied by a different group so that the supposed saving on which re-investment choices are predicated might never appear' (III:5). A cut in one place might impose a new burden or cost elsewhere in the organization. These inter-related effects often go unrecognized: 'people don't get what it costs the system' (III:7). For instance, it was perceived that many clinicians do not think about what their service costs the system when they provide care. So how might priority setting processes ensure that these system-wide impacts are identified and incorporated (III:6)? As one participant stated, "You may not know until 5-10 years down the road if the choices that seemed equivalent today really are so". (II:4) Such an observation argues for longitudinal follow-up and evaluation studies.

Finally, there was interest from a number of participants in determining the relevant strengths and weaknesses of across the board versus incremental approaches to the introduction of new formal priority setting processes. 'Is there enough of a body of evidence as to whether 'big bang' implementation or the accumulation of micro-trials is more effective at changing organizational decision making systems and patterns' (III:9)? What is a good 'entry point' for introducing an explicit priority setting framework in an organization (I:9)? Again, comparative research might profitably examine this in some detail.

#### Priority setting implementation

Choosing among options requires decision criteria with adjudicative power; that is, they can distinguish among different funding proposals on the basis of the features which decision-makers value. Participants identified a number of issues faced and where better criteria would be beneficial. In particular there were questions about how to give due consideration to strategic investments (I:5), performance agreements (II:7), long-term or transformative changes (II:4), and non-health outcomes (I:11). One suggestion was to study criteria already used in formal priority setting processes with a sort of 'sensitivity analysis' (I:6)--that is, to test how different groups within the organization (e.g., program managers versus senior executive members) understood and weighted different levels of a given rating scale employed in determining overall benefit of specific proposals. Some respondents wondered if there was any prospect of developing a 'criteria dictionary' (I:10) of measures that have been used for priority setting in other contexts and either formally validated or otherwise found useful.

Certain priority setting approaches, such as PBMA, necessarily link questions about investment and disinvestment of services; however, participants wondered what was known about the way in which decision-makers understand and interpret these two ideas. Do views about disinvestment as a concept differ from thinking about new investment (III:5)? While "everyone is there [when there's a chance] to spend money" (II:9) comparatively little thought seems to go into plans for reducing or eliminating spending. Often the participants propose a 'hand-grenade' option (II:9)--blowing up a service in a way that is, and is known by all to be, unrealistic (such as stopping all surgeries for the balance of a fiscal year). In many cases, program managers want the ability to pair investment and disinvestment proposals - 'if we cut X, we can do Y' (III:3)--and so retain any freed-up resources within their own program. A disinvestment is seen as 'taking something away' from someone--perhaps community groups or other interests acquire a 'sense of entitlement' to existing services or programs. This issue might be circumvented when the question is whether or not to invest in new initiatives which do not (or may not) have an established and organized constituency (III:5). The problem may be exacerbated if disinvestment and new investment are not directed to the same target populations. Many participants wondered about the importance of terminology; for instance, would a process be more easily accepted if a term like 'resource re-allocation' was substituted for 'disinvestment' (I:5)?

One forum participant noted that "how one bundles trade-offs is quite fascinating" (II:5). The way in which options are framed or linked can most definitely affect the way in which choices are justified. These effects are most easily seen where the resource allocation decisions involve global rather than earmarked or targeted sources of money. Research with those doing health care priority setting should be focused on helping to understand the extent to which these positions may be pre-planned. Alternatively, positions evolve during the course of negotiations as a response to the understood political influence and power of the different players in the priority setting 'game'. When research into priority setting processes looks only at what happens around the 'decision table' it may miss important questions about how different options are reached by individual departments or portfolio managers and how the choices are 'filtered' prior to that point. One health authority representative noted that in their experience, each portfolio was limited to three submissions--but it was unclear what processes narrowed the field down to these final options (I:9). In other words, 'how do we come up with the areas considered for investment or disinvestment--out of the whole range of things that might be possible candidates' (III:8)? Are the right options on the table at all? Research efforts to explore these questions would require deeper and more extensive engagement with decision-makers at all levels of an organization.

Some participants noted that those setting priorities had to manage conflicting role loyalties. 'How could you be sitting at the table and not get anything for us?' is something that those who are involved in organization-wide priority setting hear from their colleagues when they return to their home departments (I:9). Executive members themselves speak to these dilemmas: as one stated, "I know I need to defend my program, but how do I fit into the organization?" (II:6). There are also issues for clinicians who must balance roles as patient advocates with functions as gatekeepers to the system and its resources (II:1). Particularly in smaller sectors or communities, priority setting choices cannot simply be seen in the abstract--decision-makers know that their selections will impact identifiable individuals, even friends or neighbours (II:8). 'It's not just a position but a name' (III:6). There seems to be considerable scope to study individual stories about how decision-makers respond to these pressures and expectations, and strike a balance with their understanding of supposed organization-wide needs.

In the eyes of the decision-makers participating in the forums, effective priority setting and resource allocation is often held back by a lack of good, appropriate, applicable, reliable and valid data (III:6). There seemed to be a general consensus that more effort in local data collection (as well as improved local access to data collected for provincial performance reporting purposes) would produce a solid return on investment (III:7).

Taking a step back, participants recognized that health care managers have not frequently engaged in use of a formal, explicit framework for priority setting and resource allocation. Forum participants wondered if members of their own organizations had the skills and capacities needed for this work (I:9)--and wondered if research could help to systematically identify what these skills and capacities might be. Investigation of the sorts of organizational structures and supports that would best assist staff in these efforts was felt to be equally important (I:9; I:11). Health authorities are being asked to make important resource allocation decisions, and so one might wish to know if their current organizational forms are "fit for purpose" (III:9).

Finally, health system managers are under pressure from many directions to increase the extent of public engagement in decision making. This was clearly on the minds of participants who raised various questions around obtaining public input such as, 'what is its purpose?' and, 'how should it be used?' (I:6) i.e., how might the public's views and values be integrated with other forms of evidence? Public engagement efforts can be time-consuming and costly, so decision-makers asked whether evidence based guidance can be developed and suggest when it is worthwhile or necessary. Is there a way to prioritize the decisions that are subject to formal public participation efforts (II:3, II:11)? To what extent can or should the public be engaged as questions get to be more narrow and 'technical' in nature (I:6; II:11)? One purpose of public engagement may be to test whether or not decision-maker assumptions about what the public wants are in fact accurate and valid (II:10).

## Discussion

In recent years there has been a good deal of research into health care priority setting [19-27]. Some findings are clear. Priority setting is more than a technical exercise; it needs to be understood as a management process [28]. Economic approaches to priority setting should incorporate ethical principles and vice versa [16,29,30]. Both researchers and decision-makers need to think broadly about what constitutes appropriate and relevant evidence [31,32]. The literature suggests that there are several gaps in knowledge, such as the evaluation of priority setting frameworks relevant to health outcomes and other interim outputs (e.g., does formal priority setting help with setting useable priorities; are these priorities used to make decisions; does a formal framework increase the use of evidence?) and in terms of appropriate ways to engage the community in resource allocation decisions

Given this work and the myriad of challenges faced by decision-makers, we believe that future research in this area should be highly collaborative. The exact questions must be shaped as much by decision-maker input as researcher interest. The current project aimed to develop a set of research priorities in the field of health care priority setting. While it is not our intent to suggest that the priorities identified are all encompassing, nor necessarily apply in all jurisdictions, our experience in this field both within and outside of Canada would suggest that the issues that were raised should be relevant for most jurisdictions faced with allocating a finite set of resources.

The forum process resulted in a research agenda with the following characteristics: (1) The province-wide nature of the project allowed us to identify a comprehensive range of issues, including those most relevant for organizations at various stages of development in formal priority setting and resource allocation work, while recognizing different geographies, populations and health needs, organizational structures, service mixes, financial positions, etc; (2) There is considerable potential and desire for comparative work, which would allow health regions to share experiences and avoid 'reinventing the wheel'; (3) We have identified priorities that already have decision-maker buy-in, so it should be somewhat simpler and quicker to promote subsequent dissemination and uptake in British Columbia.

Several other general desirable outcomes were obtained from the very process of engaging in these forums. To begin with, they demonstrate one successful way of bridging academic and practice worlds. Principles identified in the knowledge transfer and exchange literature--such as two-way interaction among decision-makers and researchers working together to evolve priority setting practice-were demonstrated [2-4]. We speculate that this will result over the longer-term in a vibrant and growing network of BC researchers focused on priority setting, as well as a more common understanding of formal priority setting approaches and application of key principles at the health authority level. Such embedded knowledge should then contribute to improvements in routine priority setting practice.

The process allowed for sharing and networking among the health authorities themselves. Health authority personnel who attended were informed during the course of the forums about current priority setting research and practice in BC - their attendance increased their awareness of frameworks in use and likely promoted more in-depth contemplation regarding priority setting in the province. This form of research, involving active and reflective engagement in priority setting exercises, is also conducive to organizational learning and creation of greater awareness and understanding among decision-makers about how choices affect the organization as a whole.

Our observations suggest that the challenges, ideas and research topics would not have arisen - at least in the form they did - without direct interaction between researchers and decision-makers. Even as applied health services researchers, working closely with decision-makers in health service organizations, we could not predict, nor would we presume to know, the intricacies of priority setting at the coal face. We also observed a high level of peer to peer interaction between decision-makers from different health authorities. This was mentioned in the participant evaluations as a valuable aspect of the project. Furthermore, the expenditure on this team planning exercise was relatively modest, amounting to $37,500 total.

Would we do anything differently? We offer two suggestions.

• First, asking decision-makers to give up three half days within 9 months was in hindsight asking too much. If we were to do this again, we would have one day-long workshop geared towards a facilitated group discussion to maximize peer-to-peer and researcher-to-decision-maker interaction. If more then 20 decision-makers were interested in attending, we would hold separate workshops but ensure that all health authorities were represented at each session.

• Second, in follow-up, we would suggest having a more formal process in place to engage decision-makers with transition from an idea to an actual research question and, in due course, to a full research proposal. With the BC geography this is perhaps difficult, but nonetheless, allocating the budget to ensure one-on-one meetings with each of the health authorities following a primary workshop would in our view result in greater likelihood of ongoing research collaboration.

A few other potential limitations are worth mentioning. Were the 'right' people involved in the forums? Moving research forward effectively in practice settings requires a good balance between people who can speak to the technical issues 'at the coal face' of priority setting and resource allocation - those who know the challenging issues and dilemmas firsthand -- and those who are senior leaders able to devote resources to research. In this regard, our participants represented a good balance. In terms of previous experience, some participants had much direct priority setting experience to cite, others had relatively less or none. Most participants seemed actively engaged, though not all and not consistently. The fact that many participants returned for subsequent forums speaks to their engagement and sustained interest in this work, especially given the many competing demands on decision-makers' time. A conscious choice was made not to include personnel from the Provincial Ministry of Health; the focus was squarely placed on regional decision making.

We did not seek consensus, where all participants necessarily agreed upon particular research priorities shared in all health regions. Rather, the directions reported here reflect the whole range of topics raised; some persons and regions may be more interested in some of these than others. We have not reported here every possible research question that was raised during forum discussions; instead we have tried to group them into broad theme areas, with the attendant risk of omitting details which might be potentially very important to individual participants. Finally, the health authorities in BC have different populations and geographies. We wanted to be sure that the final agenda reflects this range of interests. Thus, we must ask whether or not some participants dominated the discussion and the outcomes. In careful review of the forum notes, most individuals did contribute; less vocal participants were called out for their perspective. However, it is realistic to suggest that some participants had a deeper understanding and greater engagement with the matters being discussed and their specific views may have risen to the top more readily.

Participants had several opportunities to validate the findings of this research; thus we are confident that the priorities represent their immediate needs and interests. The depth of that interest, however, will be shown by whether or not successful research collaborations are subsequently pursued. Of course, research proposals will necessarily be fitted to or constrained by available funding streams. Finally, there is frequent turnover among decision makers in the health care sector (already including some of our partners in this project). If the priorities we have identified are truly those of the health delivery organizations, rather than of the particular participants, then they should survive such developments intact. Again, time will tell.

## Conclusion

These forums have given us insight into what decision-makers see as important, and have uncovered numerous areas where, jointly, research questions can be posed. We were able to validate the importance of our starting point as well as to observe the emergence of additional concerns and directions of critical importance to these decision-makers at this time. While some, indeed probably most, of the research priorities are likely relevant elsewhere, we would advocate for others in different contexts to undertake their own research priority generating exercise. Providing an environment where researchers and decision-makers can interact, debate and collaboratively generate a set of research directions should be seen as a positive step towards the goal of a more efficient and sustainable health care system.

## Competing interests

The authors declare that they have no competing interests.

## Authors' contributions

CM, SP and SM were project Investigators and conceived and prepared the successful funding application. All authors attended the three Team Planning Forums. NS and EC conducted the data analysis. NS prepared the initial manuscript draft. All authors reviewed, commented upon and approved the manuscript.

## Pre-publication history

The pre-publication history for this paper can be accessed here:


